# Virus-Induced Gene Silencing, a Post Transcriptional Gene Silencing Method

**DOI:** 10.1155/2009/198680

**Published:** 2009-06-15

**Authors:** Turgay Unver, Hikmet Budak

**Affiliations:** ^1^Biological Sciences & Bioengineering Program, Faculty of Engineering and Natural Sciences, Sabanci University, Orhanli, Tuzla, Turkey; ^2^Kocaeli University, Arslanbey MYO, Izmit, Turkey

## Abstract

Virus-induced gene silencing (VIGS) is one of the reverse genetics tools for analysis of gene function that uses viral vectors carrying a target gene fragment to produce dsRNA which trigger RNA-mediated gene silencing. There are a number of viruses which have been modified to silence the gene of interest effectively with a sequence-specific manner. Therefore, different types of methodologies have been advanced and modified for VIGS approach. Virus-derived inoculations are performed on host plants using different methods such as agro-infiltration and in vitro transcriptions. VIGS has many advantages compared to other loss-of-gene function approaches. The approach provides the generation of rapid phenotype and no need for plant transformation. The cost of VIGS experiment is relatively low, and large-scale analysis of screening studies can be achieved by the VIGS. However, there are still limitations of VIGS to be overcome. Nowadays, many virus-derived vectors are optimized to silence more than one host plant such as TRV-derived viral vectors which are used for *Arabidopsis* and *Nicothiana benthamiana*. By development of viral silencing systems monocot plants can also be targeted as silencing host in addition to dicotyledonous plants. For instance, Barley stripe mosaic virus (BSMV)-mediated VIGS allows silencing of barley and wheat genes. Here we summarize current protocols and recent modified viral systems to lead silencing of genes in different host species.

## 1. Introduction

Gene silencing at posttranscriptional level, posttranscriptional gene silencing (PTGS), is an RNA-mediated systemic silencing mechanism which was described as quelling in fungi [1] and RNA interference in animals [2]. To specifically silence or knock down the expression of targeted gene in plants several approaches of PTGS have been developed. Virus-Induced Gene Silencing (VIGS) is one of these tools to suppress expression level of the gene of interest in plants [3, 4]. The term VIGS was first coined by A. van Kammen to describe the resistance event against viral infection [5]. Plants infected by many viruses induce RNA-mediated defense which targets viral RNAs and any transgene RNA products inserted into it [6]. As a gene silencing method VIGS has several advantageous such as fast, transient suppression of gene expression, and it involves cloning of short sequence fragments of targeted gene to be silenced. As a reverse genetic approach VIGS provides silencing of target gene in sequence specific manner. RNA-induced gene silencing mechanism is also acting on VIGS in which 21–25 nucleotide sequence of small interfering RNAs (siRNAs) guides specific cleavage or suppression of target mRNAs at posttranscriptional level [2, 7]. siRNAs which are processed from long double-stranded RNAs (dsRNA) by DICER, an RNAse-like enzyme, are then incorporated into RNA-induced silencing complex (RISC). This complex with siRNA targets specific mRNA transcripts having sequence complementarity with the specific siRNA. In other words the antisense strand of the siRNA associates with the RNAi silencing complex (RISC) to target homologous RNA for degradation [8]. dsRNAs may be originated in infected plant during cytoplasmic replication of positive-sense single-stranded (ss)RNA viruses and in the case of replicative form and replicative intermediates may represent the pool of dsRNAs [6]. For transgenes dsRNA may be generated by host RNA dependent RNA polymerases (RdRp) [9]. To be a PTGS inducers transgenes also designed and constructed to produce dsRNA [10].

## 2. Development of VIGS Methodology

Some virus species were previously modified and used for silencing the gene of interest (Table 1). Tobacco mosaic virus (TMV) is one of the modified viruses which was used for effective *pds* gene silencing in Nicotiana benthamiana plants [11]. TMV is the first modified virus for application of VIGS methods to plants. The viral delivery leads downregulation of transcript of target gene through its homology dependent degradation so potential of VIGS for analysis of gene function was easily recognized [3]. Thomas et al. detected the minimum length of RNA for PTGS. A minimum of 23 nucleotide possessing 100% homology to the target gene was observed to be required but not enough for efficient PTGS, and longer identical sequence is needed to initiate silencing [12, 13]. Tobacco rattle virus (TRV) was also modified to be a tool for gene silencing in plants. VIGS has been effectively applied in *N. benthamiana* [14] and in tomato [15] by using TRV vectors. The significant advantage of TRV-based VIGS in *Solanaceous* species is the ease of introduction of the VIGS vector into plants. The VIGS vector is placed between Rigth Border (RB) and Left Border (LB) sites of T-DNA and inserted into *Agrobacterium tumefaciens* [15, 16]. Another property of TRV is the more vigorous spreading all over the entire plant including meristem, and infection symptoms of TRV are mild [15]. Modified TRV vectors such as pYL156 and pYL279 have strong duplicate 35S promoter and a ribozyme at C-terminus for more efficient and faster spreading. These vectors are also able to infect other plant species [13, 14]. TRV-based vector has been used by Liu et al. for gene silencing in tomato [14]. Dalmay et al. have also used TRV-based VIGS to silence gene in *A. thaliana* [9]. Burch-Smith et al. [17] have developed an efficient TRV-based VIGS method to silence the *A. thaliana* genes with minimal modification of widely used TRV-based VIGS technique. Very recently, Pflieger et al. [18] have shown that a viral vector derived from Turnip yellow mosaic virus [TYMV) has the ability to induce VIGS in *Arabidopsis thaliana*. VIGS of *N. benthamiana* using Potato virus X (PVX) was also achieved [19]. PVX-based vectors have more limited host range (only three families of plants are susceptible to PVX) than TMV-based vectors (nine plant families show susceptibility for TMV) but PVX-based vectors are more stabile compared to TMV [20]. 

Geminivirus-derived vectors can be used for VIGS studies especially to study function of genes involved in meristem function. Tomato golden mosaic virus (TGMV) was used to silence a meristematic gene, proliferating cell nuclear antigen (PCNA) in *N. benthamiana* [34]. The TGMV-based silencing vector had been used for also silencing of nonmeristematic gene silencing [39]. Satellite-virus-based vectors are also used for efficient gene silencing in plants only with the help of other helper viruses. This two-component system is called Satellite-virus-induced silencing system, SVISS. In a study Tomato yellow leaf curl China virus being helper and a modified satellite DNA ware used to silence gene in *N. benthamiana* [38]. There are other viruses modified for silencing of dicotyledonous plants such as *African cassava mosaic virus* in cassava [37], *Pea early browning virus* in pea [29], and *Bean pod mottle virus* in soybean [28]. 

Previously barley stripe mosaic virus (BSMV) was developed for efficient silencing of *pds* gene in barley [26]. This system was then used for silencing of wheat genes [27]. BSMV is a positive sense RNA virus containing a tripartite (*α*, *β*, *γ*) genome. The modified *γ* of BSMV genome replaced by DNA vector was used for plant gene cloning. *β* genome has been deleted for viral coat protein production defect. Each of the modified DNAs is used to synthesize RNAs by in vitro transcription. Recently, Brome mosaic virus strain has been modified for VIGS of *pds*, *actin,* and *rubisco activase*. These genes were also silenced in important model plants such as rice [33].

## 3. Methods Used in VIGS

### 3.1. PVX (Potato Virus X)-Derived VIGS for Potato Silencing

PVX is RNA virus and infects broad range of solanaceous plants. A PVX derivative vector, an agroinfection vector, pGR106, has been previously constructed for gene silencing [19]. The vector was also used for the PVX-mediated VIGS in leaves and tubers of potato plants [21].

#### 3.1.1. Construction of PVX-Derived Vectors

PVX.GFP and PVX.PDS_AS_ can be constructed via PCR-based cloning using specific oligonucleotide primers incorporating *Asc*I and *Not*I restrictions sites, respectively, at the 5′- and 3′-termini into pGR106, a PVX derivative vector (Sainsbury Laboratory, Norwich, UK).

#### 3.1.2. Agrobacterium Tumefaciens Transformation

Transformation procedure can be followed as outlined previously [40]. *A. tumefaciens* strains (such as LB4404 and GV3101) should be prepared, and 500 mL of SOB medium (2% Bacto tryptone, 0.5% Bacto yeast extract, 10 mM NaCl, 2.5 mM KCl) in a flask should be inoculated with 1.0 mL of an overnight culture of bacteria for 6 hours at 28°C with shaking till OD550 reaches 0.7. The culture then chilled on ice for 30 minutes. The cells should be harvested at 6000 rpm for 10 minutes at 4°C. The pellet will be washed four times with 200 mL 10% glycerol (90% sterile water). The final re-suspension can be made with 0.5 mL in ice cold 10% glycerol. The prepared competent cells can be used immediately or stored at −80°C in small aliquots. Transformation of electrocompetent *A. tumefaciens* cells is performed by an electroporator. A prechilled electroporation cuvette is filled with 20–30 *μ*L electrocompetent cells and up to 5 *μ*L ligation products and should be treated with recommended 330 *μ*F capacitance, 4000 Ω resistance, and 380 V^1^ voltage. Cells are then put into 0.5 mL of SOC medium and incubated for 1 hour with shaking (100 rpm). The transformed cells are selected via antibiotic selection on spread plates with supplemented selection [40].

#### 3.1.3. Agrobacterium Infection of Plants

*Agrobacterium tumefaciens* strain possessing helper plasmid pSoup is generally transformed with PVX.GFP or PVX.GOI using procedure described above. Agroinfiltration of *N. benthamiana* and Solanum species should be performed as follows. PVX.GOI construct containing *A. tumefaciens* culture will be grown overnight at 28°C, harvested at 3000 rpm for 20 minutes, and resuspended in the same volume of 10 mM MgCl_2_, with 100 *μ*M acetosyringone and 1 mM Mes, pH 5.6. The culture should be infiltrated into leaves by a syringe at lower face [40].

### 3.2. TRV-Derived VIGS for Arabidopsis Silencing

**Figure 1:**
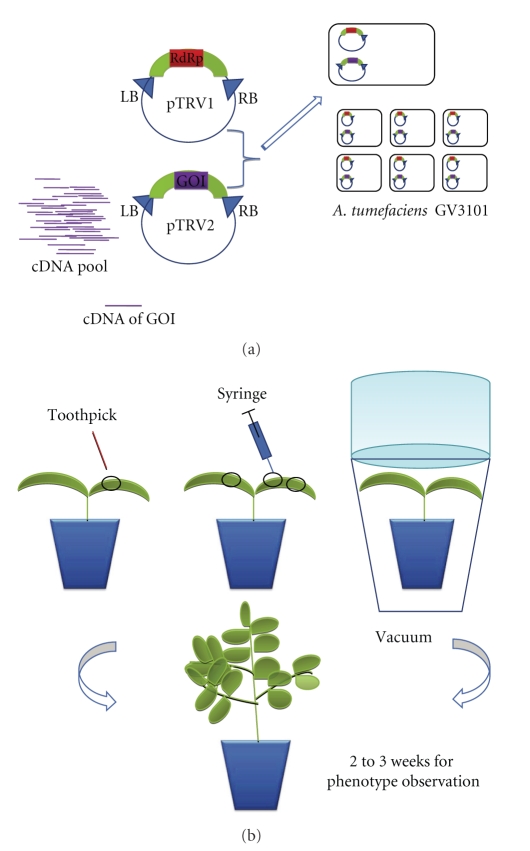
TRV-mediated VIGS in *N. benthamiana*. TRV-based virus induced gene silencing assay covers many steps; the gene with known sequence is first selected and then genetically engineered for cloning into pTRV2. pTRV1 consists of a TRV1-based cassette (RNA-dependent RNA polymerase gene, movement protein, etc.), LB and RB site for plant transformation. The plasmids are transformed into *A. tumefaciens*, and then agro-inoculation is applied. *Agrobacterium* can be inoculated on plant into seedling by a toothpick, a syringe and a vacuum infiltration as shown in the picture.

The most widely used viral delivery vectors are Tobacco rattle viruses (TRV, 16] because introduction of virus into plant including is easy in meristematic tissue [16]. TRV-mediated gene silencing was applied to many plants from diverse genera such as *Nicotiana benthamiana* [14, 16], tomato [15], pepper (*Capsicum annuum*; 32), potato (*Solanum tuberosum*; 33), and petunia (*Petunia hybrida*; 34) from Solanaceae family, opium poppy (*Papaver somniferum*) from Papaveraceae [23], and *Arabidopsis thaliana being* a model organism [17]. The TRV silencing in plants is usually mediated by *Agrobacterium tumefaciens.* TRV vectors pTRV1 and pTRV2 are placed between LB and RB sites separately. One of these vectors pTRV1, is constructed with GOI for targeted gene silencing (Figure 1). 

#### 3.2.1. Construction of TRV Vectors and Agrobacterium-Mediated Infiltration

The TRV vectors pTRY1 (pYL192) and pTRY2 (pYL156) have been described earlier [14], and the procedure can be followed described by Birch-Smith et al. [17]. *Xba*I-*Eco*RI-cut pTRV2 vector is ligated with *Xba*I-*Eco*RI-engineered PCR fragment of GOI and then transformed into *A. tumefaciens* GV3101 strain which is made electrocompotent (described in Section 3.1.2). The *Agrobacterium* culture transformed with both pTRV1 and pTRV2-GOI (grown in 50 mg/L gentamycin and 50 mg/L kanamycin overnight culture) and infiltrated into *Arabidopsis* leaves by pressing a syringe (described in Section 3.1.3, Figure 1).

### 3.3. “One-Step” TYMV-Derived Arabidopsis Silencing

Turnip yellow mosaic virus is a positive strand of RNA virus from the genus Tymovirus and infects many *Brassicaseae* including *Arabidopsis* [41]. Recently, Pflieger et al. [18] have developed a TYMV-derived vector to induce VIGS in *Arabidopsis.* The TYMV-derived vector for efficient silencing includes inverted repeats of target gene fragments. The system has ability to silence the gene even expressed in meristem and contains only a single vector. The other advantage of the TYMV mediated VIGS system that allows direct delivery of plasmid DNA to plant cells using rub-inoculation is the precluding of in vitro transcription, biolistic, and agroinfiltration steps [18]. 

#### 3.3.1. Cloning of Plasmid DNAs

The plasmid pTY has been generated by Pflieger et al. [18] using full-length TYMV cDNA clone under the control of the duplicated CaMV 35S promoter and terminator. This vector can be used for efficient gene silencing by cloning the gene(s) of interest into the vector. For example, pTY-PDS52-IR can be obtained by cloning the self-hybridized palindromic oligonucleotides PDS52 into the *Sna*BI site of pTY-S.

#### 3.3.2. Preparation and Transfection of Protoplasts

Protoplasts of *A. thaliana* can be prepared from cell suspension culture using the procedure described by [42]. A total of 106 protoplasts are transfected DNA plasmids (prepared as in Section 3.1), using the quantities indicated. Transfected protoplasts are incubated at 24°C in the dark for 48 hours (18).

### 3.4. Barley Stripe Mosaic Virus (BSMV)-Mediated Silencing

**Figure 2:**
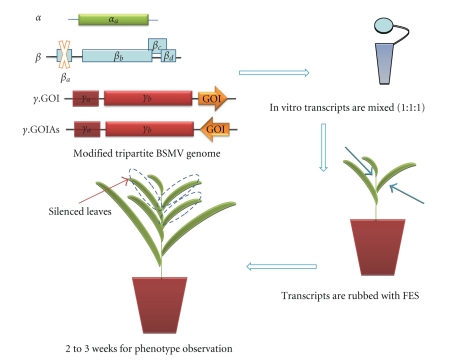
BSMV-mediated VIGS in barley. Barley stripe mosaic virus has a tripartite genome, and it has been modified to specific VIGS in barley plants [26, 27, 43].

The p*γ*.bpds4As can be used to make construction as p*γ*.(gene of interest, GOI)As by replacing pds4 insert with short GOI fragment applying restriction digestion. The same procedures can be followed for p*γ*.(gene of interest, GOI)S silencing using p*γ*.bpds4S as template [26] (Figure 2).

#### 3.4.1. Barley and Wheat Pds Gene Silencing and Measurement of Silencing Levels

**Linearization of Plasmids**

For linearization, p*α*, p*β*Δ*β*a, p*γ*, p*γ*.bpds4S, and p*γ*.bpds4As plasmids should be digested with following restriction enzymes. p*α* plasmid DNA is digested with *Mlu*I enzyme. To perform digestion, 10 *μ*g purified p*α* plasmid DNA, 1X RE buffer, 10 U *Mlu*I enzyme, and PCR grade water are combined in a sterile eppendorf tube to a final volume of 50 *μ*L. Mixture is incubated at 37°C for 2 hours. *Bcu*I enzyme can be used for p*β*Δ*β*a plasmid DNA digestion. For digestion, 10 *μ*g purified p*β*Δ*β*a plasmid DNA, 1X, 10 U *Bcu*I are combined in a sterile tube to reach a final volume of 50 *μ*L PCR water is used. Mixture is incubated at 37°C for approximately 2 hours. p*γ* plasmid can be digested with *Bss*HII enzyme. To generate linearization of p*γ* vectors 10 *μ*g p*γ* plasmid DNA, 1X enzyme buffer, 10 U *Bss*HII enzyme, and PCR grade water are combined in a tube to handle a final volume of 50 *μ*L. Mixture is generally incubated at 50°C for 2-3 hours. After the incubation samples should be observed on 1% agarose gel. Linearized plasmids should then be excised and purified [26, 27, 43].

**In Vitro Transcription**

In vitro transcription is performed for the silencing of selected target gene. It requires at least three separate in vitro transcription reactions which are the transcription of *α*, *β*Δ*β*a, and *γ* linearized genomes. According to manufacturer's procedure mMessage mMachine T7 in vitro transcription kit (cat no: 1344, Ambion, Austin, TX) transcriptions are performed. Components are mixture in a sterile tube: separately for each linarized plasmids (*Mlu*I digested p*α*-*Bcu*I digested p*β*Δ*β*a, *Bss*HII digested p*γ*- or *Bss*HII digested p*γ*.bpds4S and *Bss*HII digested p*γ*.bpds4As) 80 ng template is used per one silencing reaction (linearized plasmid DNA), 1X Buffer (Ambion), 1X nucleotide mix with NTP Cap (Ambion), 0.3 *μ*L of T7 RNA polymerase mix (Ambion), and sterile distilled water are combined up to 3 *μ*L. Mixture is incubated at 37°C for 2 hours and stored at −80°C until use [26, 27, 43].

**BSMV Transcript Inoculations on Plants**

Barley and wheat plants can be used for BSMV-mediated PTGS. The second leaves (approximately 7–10 days upon germination) should be inoculated with BSMV for silencing. All BSMV transcripts which are *α*, *β*Δ*β*a, and *γ* will be mixed in a 1 : 1 : 1 ratio (1.0–1.5 *μ*g of each transcript concentration is observed on spectrophotometer, Figure 2, Table 2). Transcription mix is combined with 50 *μ*L FES. 50 mL FES requires GP solution (10X GP: (18.77 g glycine, 26.13 g K2HPO4, ddH_2_0 upto 500 mL, sterilized by 20 minute autoclaving) which is then combined with 2.5 g sodium pyrophosphate, 2.5 g bentonite, 2.5 g celite with ddH_2_0 up to 250 mL and re-autoclaved [44], and directly applied to the second leaf (when it is 5–7 cm long) from the bottom of leaf to the tip. After 7–10 days post inoculation (dpi), appearance of mosaic symptoms on leaves should be observed showing systemic spread of the virus. Leaves from inoculated plants are collected after approximately 14-15 day postinoculation (dpi) in order to check *pds* gene silencing level by qRT-PCR [26, 43].

## 4. Improvements of Virus-Induced Gene Silencing

Gene specific silencing via VIGS system is now used for diverse monocot and dicot plant species. Therefore, a number of viral-derived vectors have been developed (Table 1), and many procedures have been optimized by the researchers. TRV system was efficiently optimized for efficient silencing of Solanaceous plants [14, 15], and the system was also applied for tomato to study role of fruit ripening genes [45]. TRV-mediated VIGS has been modified for robust and effective gene silencing in a model organism, *Arabidopsis* by [17]. The emerging model plant columbine *Aquilegia vulgaris* has been efficiently silenced via TRV-mediated VIGS [24]. Many economically important plants were studied to optimize TRV-derived VIGS silencing such as opium poppy [23]. Efficiency of the TRV-derived viral vector used VIGS system on tomato fruit *via* agro-injection has been improved up to 90% silencing compared to agro-infiltration of cotyledons and first leaves of plants (66%) [46]. Lacomme et al. [47] have described a method to enhance the robustness of the VIGS phenotype by increasing the level of dsRNA by incorporation of 40–60 base direct inverted-repeats into a plant viral vector. Cheapness and easiness of *Arabidopsis* silencing have been improved *via “one-step*”* TYMV*-derived VIGS [18]. Monocot plants are also subjected to be silenced *via* VIGS. For this propose, Holzberg et al. [26] developed a BSMV-mediated VIGS system for barley, and Scofield et al. [27] have applied the system to wheat. BMV has also been used to silence genes in monocot plants. Ding et al. [33] efficiently silenced the genes in barley, rice and maize.

## 5. Comparison of VIGS with Other Gene Silencing Methods

VIGS has many advantages and disadvantages compared to other techniques used for functional analysis of plant genes. Generally, the method is chosen for its reliability, low cost, easiness, and rapidness. Several tools have been used for identification of loss-of-function of gene(s) such as, TILLING, chemical and physical mutagenesis, T-DNA, and transposon insertion techniques. However, VIGS presents an intended potential for the researchers working with functional genomics due to the fact that it avoids many of limitations of the above approaches. Its main advantage is the generation of rapid phenotype and that there is no need for plant transformation. The cost of VIGS experiment is relatively low; *Agrobacterium* or in vitro transcription mediated VIGS assays do not cost effectively. VIGS method also provides a large-scale screening of genes for functional analysis. Moreover, there is no need to screen large populations to detect the function of a specific gene; only a single plant is enough to follow phenotype with targeted silencing. Therefore, repeating the experiment is easy and time effective. Host range wideness of viral vectors is the other versatility of the approach. For instance TRV can infect spinach, beet, potato, and tobacco naturally. Hence TRV-based VIGS is applied to *Nicotiana benthamiana*, tomato, *Arabidopsis,* chilli pepper, opium poppy, and *Aquilegia vulgaris *(Table 1). Since it does not require plant transformation, VIGS is particularly useful on plants which are difficult or impossible to transform. Therefore, VIGS system can be applied to the genes associated with embryonic development or essential housekeeping functions in plants [33, 38]. Functional redundancy problem is overcome by VIGS application using most conserved region of the gene family [26, 27]. Despite the valuable advantages of VIGS approach, there are also limitations. One of the most important limitation is that complete loss-of-function by VIGS might not be achieved. Generally 75–90% downregulation in the expression level of the targeted gene is accomplished [18, 43, 46]. Unfortunately the low level of gene expression can be enough to produce functional protein and phenotype in silenced plant. Some of viral infections can cause symptoms on plant that might mask the phenotype caused by the phenotype. This problem might be minimized as TRV-VIGS system because of mild symptoms [14, 16]. VIGS aims to silence the specific gene, which can only be achieved by sequence specific manner so the system relays on sequence information. The approach also depends on pathogen-host interaction, so the disadvantage is that pathogen infection may manipulate host function and alter development and morphology. There should be positive control in all VIGS assays to mark the effect of viral inoculation on silenced plant. Lastly, VIGS might suppress nontargeted gene in silenced plant cell or tissue [17]. This response should be addressed before the next genomic era.

## 6. Concluding Remarks

VIGS as a reverse genetics tool for functional genomics studies presenting many advantages promises rapid generation of functional genomics even proteomics. By the progressing and completing whole genome sequencing of many important crops, VIGS approach will be widely and mostly used. Despite its great potential to extensively use, many limitations remains to be overcome. Firstly host range of viral vectors will become wider; the VIGS assays and viral vectors for model organisms such as *Arabidopsis* and rice should be well optimized. As mentioned sequence information is crucial for VIGS approach so the whole genome sequence databases and EST databases will be add great contribution of VIGS usage. With the whole genome sequence availability, *Brachypodium distachyon* (L.) Beauv., a model temperate grass species, should also be used in application of VIGS system for generation of genomics information to improve temperate crops. Large-scale screening *via* VIGS-based method to detect important and fascinating phenotypes should be performed. 

## Figures and Tables

**Table 1:** Viruses used for silencing of genes and their hosts with targeted genes are listed.

Viruse/viruse type	Silencing host species	Group	Genes silenced	Natural host species	Reference
Tobacco mosaic virus (TMV)/RNA virus	*Nicotiana *	Tobamovirus	*pds*	Tomato, squash, potato, tobacco	
	*benthamiana,*				[11]
	*nicotiana tabacum*				
Potato virus X (PVX)/RNA virus	*Nicotiana *	Potexvirus	*pds*	Potato, oilseed, rape	
	*benthamiana, *				[21, 22]
	*Arabidopsis*				
Tobacco rattle virus (TRV)/RNA virus	*Nicotiana *	Tobravirus	*Rar1, EDS1, NPR1/NIM1 pds, rbcS, *	Spinach, beet, potato, tobacco	
	*benthamiana*, tomato,				
	*Arabidopsi, *solanum species,				[14, 16, 23–25]
	chilli pepper, opium poppy,				
	*Aquilegia vulgaris*				
Barly stripe mosaic virus (BSMV) RNA virus	Barley	Hordeivirus	*pds, Lr21, Rar1, Sgt1, Hsp90*	Barley, wheat	[26, 27]
Bean pod mottle virus (BPMV)/RNA virus	*Glycine max*	Comovirus	*pds*	*Phaseolus vulgaris,glycine max*	[28]
Pea early browning virus (PEBV)/RNA viruse	*Pisum sativu,*	Tobravirus	*pspds, uni, kor, pds*	*Pisum sativum,*	
	*Medicago truncatula, *			*Phaseolus*	[29, 30]
	*Lathyrus odorata*			*vulgaris*	
Satellite tobaccomosaic virus (STMV)/Satellite virus	*Nicotiana tabacum*	RNA satellite virus	*pds, rbcS, rbcL* and various genes	*Nicotiana glauca*, pepper	[31]
Poplar mosaic virus (PopMV)/RNA virus	Poplar	Carlavirus	*gfp*	*Nicotiana *	[32]
				*benthamiana*	
Brome mosaic virus (BMV)/RNA virus	Barley, rice, maize	Bromovirus	*pds, actin 1, rubisco activase*	Barley	[33]
Tobacco golden mosaic virus (TGMV)/DNA virus	*Nicotiana *	Begomovirus	*su*	Tomato	[34]
	*benthamiana,*				
Tomato bushy shunt virus (TBSV)/RNA virus	*Nicotiana *	Tombusvirus	*gfp*	*Lycopersicon esculentum*	[35]
	*benthamiana,*				
Cabbage leaf curl virus (CaLCuV)/DNA	*Arabidopsis*	Begomovirus	*CH42, pds *	Cabbage,	
				broccoli,	[36]
				cauliflower	
African cassava mosaic virus (ACMV)/DNA virus	*Nicotiana *	Begomovirus	*pds, su, cyp79d2*	*Manihot esculenta*	
	*benthamiana, *				[37]
	*Manihot esculenta*				
Tomato yellow leaf curl China Virus (TYLCV)/DNA virus	*Nicotiana benthamiana,*	DNAbeta satellite DNA	*pcna, pds, su, gfp*	Tomato	
	*Lycopersicon esculentum,*				[38]
	*N. glutinosa, *				
	*N. tabacum*				

**Table 2:** Construction of transcripts for the BSMV inoculation [26, 27, 43].

Inoculation for silencing	p*α* transcript	p*β*Δ*β* a transcript	p*γ* transcript	p*γ*.GOIS transcript	p*γ*.GOIAs transcript	FES solution
BSMV:00 (viral control)	1.0–1.5 *μ*g	1.0–1.5 *μ*g	1.0–1.5 *μ*g	—	—	50–55 *μ*L
BSMV:GOIS (sense version)	1.0–1.5 *μ*g	1.0–1.5 *μ*g	—	1.0–1.5 *μ*g	—	50–55 *μ*L
BSMV:GOIAs (anti-sense version)	1.0–1.5 *μ*g	1.0–1.5 *μ*g	—	—	1.0–1.5 *μ*g	50–55 *μ*L
FES (non silencing control)	—	—	—	—	—	50–55 *μ*L
